# Clinical pharmacists' aspirations on prescribing in cardiology within an academic health system

**DOI:** 10.1016/j.rcsop.2026.100804

**Published:** 2026-05-23

**Authors:** Seeba Zachariah, Said Nabil, Anju Thomas, Selma Castell, Shatha Sayegh, Dixon Thomas

**Affiliations:** a College of Pharmacy, Gulf Medical University, Ajman, United Arab Emirates; b Department of Clinical Pharmacy, Thumbay University Hospital, Ajman, United Arab Emirates

**Keywords:** Pharmacist prescribing, Cardiology pharmacy, Collective autoethnography, Reflexive thematic analysis, Clinical decision-making

## Abstract

**Background:**

Pharmacist prescribing in cardiology is influenced by clinical expertise, organizational structures, and expanding scopes of practice. Although international models highlight the value of pharmacist prescribers, limited evidence describes how prescribing is experienced within cardiology pharmacy practice. This study examined the prescribing potential, contextual conditions, and motivations of clinical pharmacists as part of continuing professional development.

**Methods:**

An interview-based qualitative design informed by collective autoethnography was used to situate researchers' professional experiences and reflections. Data were derived from retrospective reflections of clinical pharmacists and a pharmacy student working within an academic health system in the United Arab Emirates (UAE). Reflexive thematic analysis, guided by Braun and Clarke's six-phase approach, was conducted with iterative reflexivity and theoretical integration.

**Results:**

Twelve themes were generated from 155 coded excerpts and grouped into three domains: what pharmacists do when prescribing, when prescribing occurs, and why prescribing is undertaken. Prescribing activities included patient assessment, dose adjustment, and initiation of evidence-based cardiovascular therapies. Key enabling conditions were specialized training, interprofessional trust, and collaborative care structures. Motivations centered on enhancing autonomy, improving timely medication optimization, and achieving meaningful patient outcomes.

**Conclusion:**

Pharmacist prescribing in cardiology is a clinically grounded and collaborative practice that suggests potential to support timely therapeutic decisions and reduces treatment inertia. Insights from this insider perspective underscore the need for structured prescribing pathways, advanced training, and supportive regulatory frameworks to enable pharmacists to practice at their full scope.

## Highlights

• Autoethnography reveals insider perspectives on cardiology pharmacist prescribing.
• Twelve themes describe what, when, and why pharmacists prescribe in cardiology.
• Prescribing activities include assessment, dose adjustment, and therapy initiation.
• Training, trust, and collaboration shape pharmacist prescribing readiness.
• Prescribing reduces treatment inertia and supports timely cardiovascular care.

## 1 Introduction

Cardiovascular diseases (CVDs) remain the leading cause of morbidity and mortality worldwide, placing increasing pressure on healthcare systems to deliver timely, evidence-based, and patient-centered care.^1^ As the complexity of cardiovascular pharmacotherapy grows, driven by expanding therapeutic options, evolving clinical guidelines, and the rising prevalence of multimorbidity, the role of clinical pharmacists has become increasingly indispensable.^2^, ^3^, ^4^ In many healthcare settings, cardiology clinical pharmacists contribute to medication optimization, risk-factor management, transitions of care, and multidisciplinary decision-making.^5^, ^6^ Their involvement has been associated with improved clinical outcomes, enhanced medication safety, and reduced healthcare utilization.^7^, ^8^

Despite this growing recognition, the scope of practice for cardiology clinical pharmacists varies widely across countries, institutions, and regulatory frameworks. Some regions grant pharmacists independent or collaborative prescribing authority, while others restrict their role to advisory functions. These inconsistencies shape not only the nature of pharmacists' contributions but also their professional identity, autonomy, and integration within cardiology teams.^9^, ^10^ Understanding how cardiology clinical pharmacists navigate these boundaries is essential for informing policy, advancing practice models, and ensuring that patients receive optimal cardiovascular care.^11^, ^12^, ^13^

Though not typically used, collective autoethnography offers a unique methodological lens through which to explore prescribing potential. By situating personal professional experience within broader cultural, institutional, and regulatory contexts, collective autoethnography allows for a nuanced examination of how prescribing authority could be enacted, negotiated, or constrained in daily clinical practice. This approach foregrounds the lived realities of clinical pharmacists capturing the tensions, opportunities, and decision-making processes that are often invisible in traditional empirical research.^14^, ^15^

Given the global movement toward expanding pharmacists' clinical roles, there is a pressing need to clarify how clinical pharmacy professionals determine what to prescribe in cardiology, when prescribing is appropriate, and where prescribing authority would be situated within the clinical workflow. Such insights illuminate the practical, ethical, and organizational factors that shape prescribing-related decision-making and can guide the development of coherent, supportive practice environments. These environments, in turn, strengthen experiential education for the next generation of pharmacists in the United Arab Emirates (UAE). For a group of cardiology clinical pharmacy professionals, envisioning future prescribing practices required grounding their reflections in their own experiential realities. The study was informed by autoethnographic accounts representing the diverse experiences of the participants. The research question guiding this inquiry was: How do clinical pharmacists identify what to prescribe, when prescribing is appropriate, and in which cardiology contexts prescribing authority would be exercised, at an academic health system in the UAE reflecting on prior internal and external experiences and observations?

## 2 Methods

### 2.1 Study design

This study employed an interview-based qualitative methodology that situates the researchers' professional experiences within broader cultural, institutional, and regulatory contexts. The terms pharmacist and clinical pharmacist are used interchangeably throughout. While not a full autoethnographic design, the study was informed by collective autoethnography to capture reflections shaped by prior experiences, organizational culture, interprofessional dynamics, and evolving scopes of practice. As practicing clinical pharmacists in cardiology, the researchers occupy an insider position that provides access to tacit knowledge, decision-making processes, and contextual nuances. Informed from prior experiences and observations at other sites and events, the participants reflected on their aspirations to prescribe at the research setting. The semi-structure interviews further enabled systematic reflexivity, theoretical engagement, and the integration of personal narrative with scholarly analysis.^14^, ^15^

### 2.2 Research setting

The study was conducted within an academic health system in the UAE, with a focus on cardiology practice. The clinical environment encompassed inpatient cardiology wards, coronary care units, outpatient heart failure clinics, and multidisciplinary rounds. Pharmacist prescribing authority is shaped by institutional policies and national regulations that delineate the boundaries of independent prescribing and collaborative prescribing. At the time of data collection in early 2026, pharmacists did not hold prescribing rights, although institutional discussions were underway regarding limited prescribing privileges for selected clinical pharmacists. Because this qualitative study was informed by collective autoethnography, the reflections extend beyond the immediate study site to include experiences drawn from multiple practice settings, professional events, and evolving guidelines related to pharmacist prescribing. These contextual factors form an essential backdrop for interpreting the researchers' experiences and reflections within this evolving professional landscape.

### 2.3 Researcher positioning and reflexivity

The researchers were clinical pharmacists and a student in training with direct responsibilities in cardiology medication management, therapeutic decision-making, interprofessional collaboration, and the training of pharmacy students. Four clinical pharmacists with varying levels of experience and one senior student in cardiology training contributed to data generation and validation of the findings. Two of the clinical pharmacists had previously completed pharmacist-prescribing training in cardiology at another hospital in the UAE, and one had developed an anticoagulation prescribing protocol for the study setting in collaboration with internal and external pharmacy and medical teams. Two other clinical pharmacists had visited and observed prescribing practice of pharmacists in the UK. These first-hand exposures to pharmacist prescribing informed their ability to reflect critically on the topic. The study was informed by collective autoethnography, as participants' reflections were grounded in their own professional experiences and continuing professional developments. Other participants had comparatively less direct exposure but were knowledgeable about pharmacist prescribing through professional networks, literature, and continuing education. This mix of perspectives enabled a balanced exploration of perceived benefits, challenges, and concerns surrounding pharmacist prescribing.

Occupying an insider role provided privileged access to the lived realities of cardiology practice, including tacit knowledge, workflow dynamics, and interprofessional interactions. However, this positionality also introduced potential biases. To address this, reflexivity was embedded throughout the research process. Researchers documented reflections on clinical encounters involving prescribing-related decisions, emotional and cognitive responses to interprofessional interactions, perceived constraints and facilitators, and ethical tensions. They also examined institutional norms and their own assumptions. These reflexive entries served as primary data and were revisited iteratively to identify patterns, contradictions, and evolving interpretations.

### 2.4 Data sources

Semi-structured interviews were used as the source of data collection. The clinical pharmacists reflected on key clinical episodes from memory, focusing on situations where prescribing decisions were central. The participants were asked in advance to be prepared (recall and review of some documents or communications) on pharmacist-prescribing before the interview. Still recall bias is possible, but the study was aiming to capture current reflections and thought process for the future than an accurate account of previous experiences. All reflections were verbal, asking prompts on experiences and observations of the participants. The participants had varying levels of experiences observing pharmacist prescribing at other sites or events, plus all of them had experiences with physicians on prescription review discussions. These reflections captured the complexity of real-world decision-making, including uncertainty, negotiation, and contextual pressures. Experiences from patient cases, team discussions, workflow constraints, and moments where prescribing authority was exercised or limited came handy in the discussion. Reflection documents were analyzed for codes and themes.

### 2.5 Ethical considerations

The study centers on the researchers' own experience and reflections. No patient or other health professional-level data, clinical outcomes, or identifiable information were included. The study adhered to institutional ethical guidelines for qualitative research involving professional reflection. Institutional Review Board approval was taken; IRB-COP-FAC-136-Mar-2025. The participants (also researchers) provided informed voluntary participation.

### 2.6 Data analysis

Data were analyzed using thematic reflexive analysis, following Braun and Clarke's iterative six-phase approach.^16^ Analysis proceeded as follows: Familiarization, transcript recordings of interviews were read repeatedly to gain a holistic sense of the material. Initial Coding, line-by-line coding was conducted to identify segments related to prescribing decisions, role negotiation, clinical judgment, regulatory boundaries, and interprofessional dynamics. Theme Development, codes were clustered into preliminary themes that captured recurring patterns, tensions, and insights. Particular attention was given to contradictions between formal prescribing authority and practical realities. Refinement and Theoretical Integration, themes were refined through constant comparison and linked to existing literature on clinical pharmacy practice, professional identity, and healthcare role boundaries. The themes were woven into a cohesive analytic narrative that integrates personal experience with broader structural and cultural interpretations. Atlas.ti version 26 was used for thematic analysis. No artificial intelligence tools were used in data analysis.

## 3 Results

**Fig. 1:**
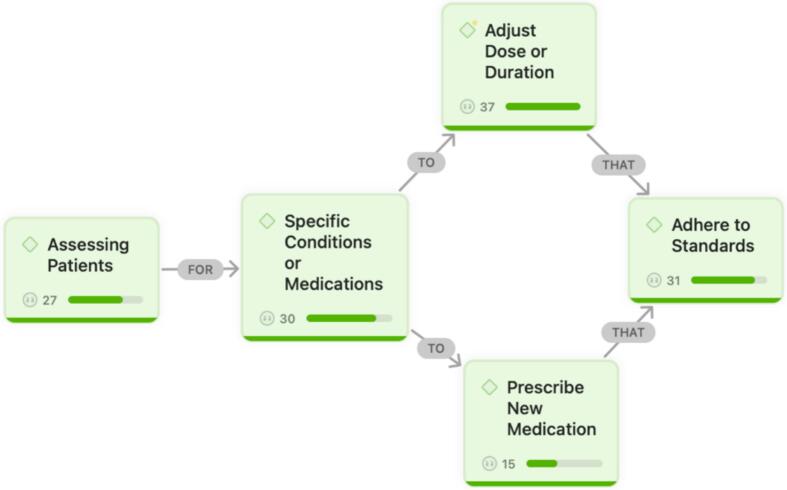
‘What’ of pharmacist prescribing.

**Fig. 2:**
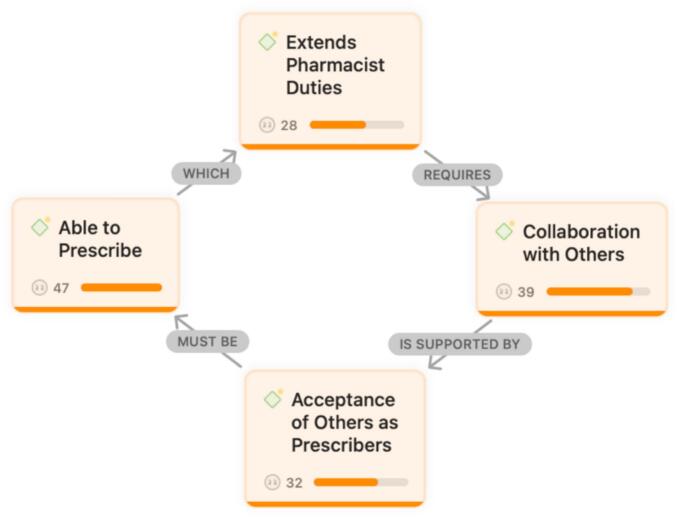
‘When’ of pharmacist prescribing.

**Fig. 3:**
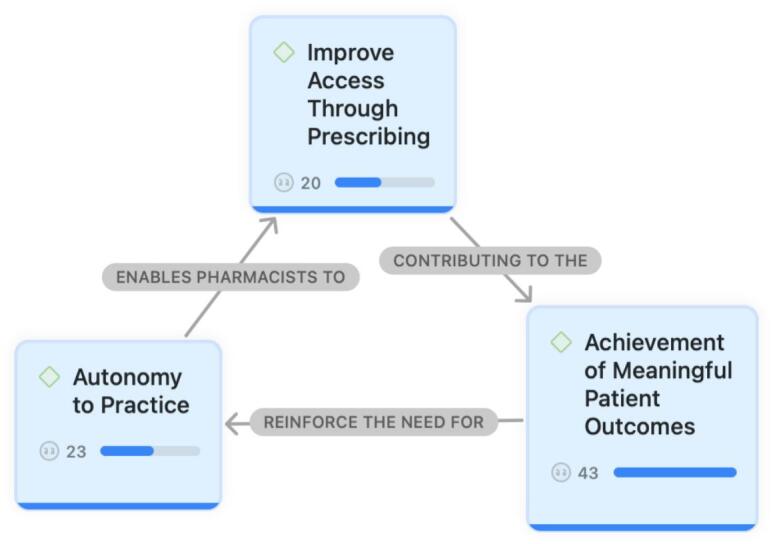
‘Why’ of pharmacist prescribing.

**Fig. 4:**
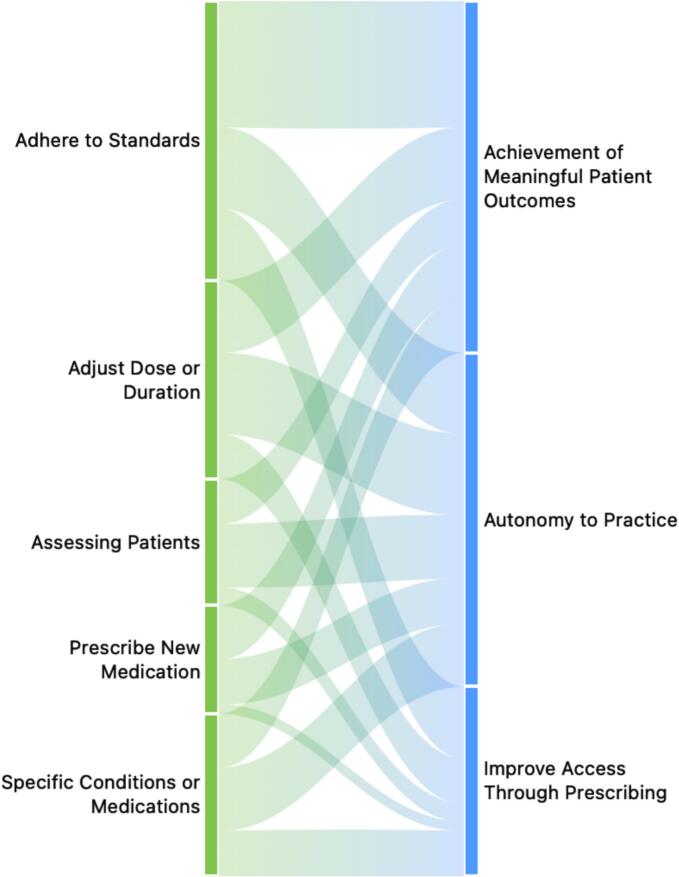
‘What’ and ‘Why’ of pharmacist prescribing.

**Fig. 5:**
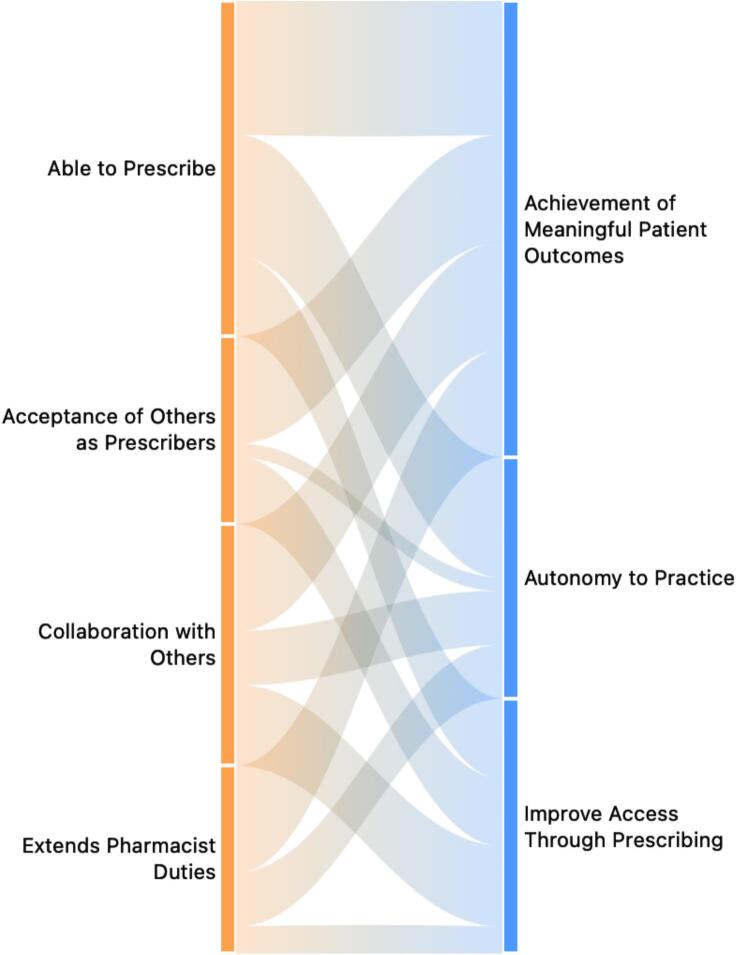
‘When’ and ‘Why’ of pharmacist prescribing.

A total of 12 themes were developed from 155 quotations in the data analyzed. Themes were categorized to ‘What’, ‘When’, and ‘Why’ of pharmacist prescribing, illustrated in Fig. 1, Fig. 2, Fig. 3. Themes showed their groundedness in quotations with associated numbers in the figures. Sankey diagrams were used to compare What and When themes with Why themes in Fig. 4, Fig. 5.

What pharmacists perceived as prescribing actions included the following themes ‘Assessing Patients’ for ‘Specific Conditions or Medications’ to ‘Adjust Dose or Duration’ or to ‘Prescribe New Medication’, that ‘Adhere to Standards’ (Fig. 1).

### 3.1 Assessing patients (Note: under each themes, relevant quotes from the interview data are provided)

“We need to consider specific patient characteristics, including comorbid conditions, before making any prescribing decision.”

“The patient's INR is monitored through point-of-care testing, and the warfarin dose is adjusted accordingly based on those readings.”

“Conditions such as hypertension, heart failure, atrial fibrillation, and dyslipidemia require continuous monitoring, regular dose adjustments, and ongoing adherence support.”

### 3.2 Specific conditions or medications

“Pharmacists who specialize in a particular area, such as cardiac medications, are better positioned to make informed prescribing decisions.”

“Pharmacists must clearly understand their scope of practice and the boundaries within which they are authorized to prescribe.”

“Our role often involves recommending or adjusting therapies such as antiplatelet agents, beta-blockers, statins, or anticoagulants for patients with acute coronary syndromes, heart failure, or atrial fibrillation.”

### 3.3 Adjust dose or duration

“For example, adjusting the warfarin dose based on the patient's INR readings is a routine and necessary part of care.”

“We frequently need to increase or reduce medication doses depending on how the patient responds clinically.”

“Therapeutic inertia has been documented in diabetes and many other chronic conditions. Some clinicians do not escalate or modify therapy when needed, and this directly affects patient progress and outcomes.”

“I would like to prescribe in heart failure because patients should be started on the four pillars of treatment, yet many of them are not titrated to the recommended target doses.”

### 3.4 Prescribe new medication

“Initiating a new medication is sometimes necessary to optimize a patient's treatment plan.”

“One of the therapies I believe pharmacists should be allowed to initiate is lipid-lowering medication for patients with dyslipidemia. Many patients with diabetes or cardiovascular risk factors would significantly benefit from starting such therapy. It is a relatively simple intervention but one that could have a substantial impact on public health.”

### 3.5 Adhere to standards

“Patients should be closely monitored according to a pre-approved plan that outlines the steps for safe prescribing.”

“In situations where a patient presents with an emergency issue, referral to a physician is, of course, necessary.”

“Many patients are unaware of their medical conditions or stop taking their medications due to concerns about side effects, which highlights the need for clear communication and monitoring.”

“All prescribing activities must be transparent. Pharmacists should maintain documentation that is clear, verifiable, and accessible for review by any authorized healthcare professional.”

‘When’ of pharmacist prescribing themes included pharmacists must be ‘Able to Prescribe’, which ‘Extends Pharmacist Duties’, requires ‘Collaboration with Others’, and is supported by ‘Acceptance of Others as Prescribers’ (Fig. 2).

### 3.6 Able to prescribe

“I have completed specialized pharmacotherapy training focused on cardiology patients. I hold board certification in cardiology, and I see around 20 cardiac patients every day. After working with cardiologists for 10 years, I have encountered a wide range of patient groups and disease presentations.”

“Only selected pharmacists who are appropriately qualified and experienced in the relevant clinical area should be granted prescribing privileges, not everyone.”

“After completing pharmacy degrees and obtaining board certification, pharmacists should work under a collaborative practice agreement with a physician who trusts their prescribing abilities.”

### 3.7 Extends pharmacist duties

“Prescribing comes naturally to more experienced staff or those who have done extensive clinical work, especially when it occurs in collaboration with physicians.”

“Pharmacists can manage medication refills without needing to refer every case back to the physician.”

“Clinical pharmacists already review prescriptions, assess patients, gather information from medical records, and evaluate therapy appropriateness. Prescribing follows the same steps. The only difference is that instead of calling a physician, the pharmacist makes the modification when it is clearly beneficial and has been previously discussed and authorized. In such cases, prescribing is simply an extension of the clinical pharmacist's existing responsibilities.”

### 3.8 Collaboration with others

“Physicians' comfort levels vary widely and often depend on their previous experience working with clinical pharmacists. In settings where pharmacists have long been integrated into multidisciplinary teams, physicians generally express strong support.”

“Nurses primarily focus on ensuring that medications are available and administered on time. Beyond that, they typically do not have negative attitudes toward pharmacist prescribing.”

“Prescribing within collaborative protocols is feasible and has been successfully implemented in many healthcare systems.”

### 3.9 Acceptance of others as prescribers

“Physicians trust clinical pharmacists and allow them to make treatment modifications, and this already happens regularly in practice.”

“Nurses usually respond positively to pharmacist prescribing because it improves workflow efficiency and reduces delays in implementing medication changes.”

“In countries where pharmacist prescribing is established, patients tend to be among the most accepting stakeholders. They appreciate pharmacists' accessibility, the time spent explaining medications, and the continuity provided in chronic cardiovascular care.”

“I feel that patients have a significant amount of trust in pharmacists.”

‘Why’ of pharmacist prescribing themes included the need for ‘Autonomy to Practice’ enables pharmacists to ‘Improve Access through Prescribing’, thereby contributing to the ‘Achievement of Meaningful Patient Outcomes’ (Fig. 3).

### 3.10 Autonomy to practice

“Prescribing rights give pharmacists a sense of ownership over patient care and enhance our professional autonomy.”

“Having prescribing authority allows us to independently make simple dose changes or initiate new medications when the evidence is clear and guidelines strongly support the decision. This avoids unnecessary steps in the healthcare system and prevents delays in the patient's treatment journey.”

“In the UK's National Health Service, independent pharmacist prescribers initiate or titrate medications such as ACE inhibitors, ARNI therapy, or anticoagulants under structured care pathways.”

### 3.11 Improve access through prescribing

“Pharmacist prescribing helps patients receive medical care more quickly.”

“Nurses work closely with clinical pharmacists, and when pharmacists have prescribing rights for selected medications, it saves time and is more convenient for everyone involved.”

“Being able to titrate beta-blockers, initiate statins, or manage anticoagulants reduces delays in care and allows physicians to focus on diagnostics and more complex cases.”

### 3.12 Achievement of meaningful patient outcomes

“Physicians will support pharmacist prescribing when they see that it adds value to patient care. The impact depends on how much value we contribute when initiating new medications or titrating existing ones.”

“Successful prescribing relies not only on pharmacological knowledge but also on effective communication and empathy.”

“Pharmacists can significantly influence cardiovascular outcomes when they are empowered to practice at the full scope of their training.”

Fig. 4, Fig. 5 illustrate how the thematic domains of pharmacist prescribing intersect within cardiology practice. Fig. 4 maps the relationship between the “What” and “Why” of pharmacist prescribing, showing how specific prescribing activities, such as assessing patients, adjusting doses, or initiating therapy connect to broader motivations including autonomy, improved access, and meaningful patient outcomes. Fig. 5 complements this by linking the “When” themes to the same set of “Why” drivers, highlighting how readiness to prescribe, collaborative practice structures, and professional acceptance align with the underlying rationale for expanding pharmacists' roles. Together, these visualizations demonstrate the coherence between pharmacists' perceived prescribing responsibilities, the conditions under which prescribing is appropriate, and the overarching goals that make prescribing valuable within cardiology care.

## 4 Discussion

Pharmacist prescribing in cardiovascular care emerged in this study as a multifaceted practice shaped by what pharmacists do, when they prescribe, and why they engage in prescribing activities. Participants described prescribing as an extension of their clinical responsibilities, particularly in assessing patients, adjusting doses, and initiating evidence-based therapies. Pharmacists' reflections also underscored that prescribing authority would allow them to act directly on clinical assessments thereby reducing delays in therapy adjustments and improving safety and reduce therapeutic inertia in cardiovascular care. These findings align with international literature demonstrating that pharmacists frequently manage chronic cardiovascular conditions by optimizing medication regimens, monitoring therapeutic response, and ensuring adherence to clinical guidelines. Studies report that pharmacists play a central role in titrating heart failure medications, managing anticoagulation, and initiating lipid-lowering therapy, reinforcing the clinical relevance of the activities identified in this study.^17^, ^18^, ^19^

The conditions under which pharmacists prescribe captured in the “when” themes highlighted the importance of competence, experience, and collaborative practice environments. Participants emphasized that prescribing authority should be granted to pharmacists with specialized training and demonstrated expertise, where advanced clinical training and credentialing are prerequisites for independent prescribing. Participants noted that physician acceptance of pharmacist prescribing is strongly influenced by prior exposure to pharmacist expertise, suggesting that trust develops through demonstrated competence within collaborative cardiology teams. The literature consistently notes that successful pharmacist prescribing models rely on strong interprofessional relationships, with physicians expressing greater trust and acceptance when pharmacists are embedded in multidisciplinary teams. This mirrors the experiences described by participants, who noted that physician comfort and support were closely tied to prior collaborative interactions.^20^, ^21^

The “why” themes further illuminate the motivations and perceived benefits of pharmacist prescribing. Autonomy to practice was seen as essential for enabling timely, evidence-based decisions that reduce unnecessary delays in patient care. This perspective is supported by studies showing that pharmacist prescribers improve workflow efficiency, reduce treatment inertia, and enhance adherence to guideline-directed therapy.^22^, ^23^, ^24^

Improved access to care emerged as another key rationale for pharmacist prescribing. Participants described how granting pharmacists the authority to prescribe select medications reduces bottlenecks, particularly in busy cardiovascular clinics where physicians must prioritize diagnostic and complex cases. The pharmacist prescribers significantly reduce wait times, streamline medication adjustments, and enhance continuity of care. Other healthcare professionals have also been shown to support pharmacist prescribing due to its positive impact on workflow and patient throughput, consistent with the experiences reported in this study.^25^, ^26^, ^27^

Ultimately, participants emphasized that pharmacist prescribing contributes to meaningful patient outcomes, provided it is grounded in strong communication, empathy, and clinical expertise. The pharmacist-led prescribing interventions improve blood pressure control, optimize heart failure therapy, and enhance anticoagulation management. Studies consistently show that when pharmacists practice at the full scope of their training, patient outcomes improve and physician satisfaction increases. Such findings therefore reinforce global evidence that pharmacist prescribing is a safe, effective, and valuable component of cardiovascular care, particularly when supported by appropriate training, collaborative structures, and clear clinical pathways.^28^, ^29^

Pharmacist prescribing also presents several challenges that must be carefully considered to ensure safe and effective implementation. Potential risks include scope creep, where prescribing responsibilities expand beyond intended boundaries, and training variability, which may lead to inconsistent clinical decision-making across practitioners.^20^, ^30^ There are also clinical situations in which pharmacist prescribing may not be appropriate, such as highly complex cardiology cases requiring diagnostic interpretation, rapidly deteriorating patients, or scenarios involving significant prognostic uncertainty.^31^ Effective prescribing models therefore depend on strong physician–pharmacist relationships, where trust, shared protocols, and clear communication pathways support safe delegation of prescribing authority. Without these relational foundations, prescribing could become fragmented or duplicative, undermining continuity of care.^32^, 33.

In the UAE context, these challenges are further shaped by the evolving regulatory landscape, heterogeneous institutional readiness, and the absence of nationally standardized prescribing frameworks for pharmacists. Variability in clinical training pathways, differences in hospital accreditation standards, and the limited number of pharmacists with advanced cardiology credentials create additional barriers to consistent implementation. Moreover, prescribing authority remains unfamiliar to many stakeholders in the UAE, making professional acceptance, role clarity, and interprofessional trust particularly critical. As institutions explore limited prescribing privileges for selected clinical pharmacists, these contextual factors highlight the need for structured governance, competency-based credentialing, and phased implementation strategies tailored to the UAE's health-system priorities.^34^, ^35^, ^36^

The insights generated from this study have perceived implications for academic health systems seeking to expand pharmacist prescribing roles. The findings suggest that prescribing privileges should be linked to advanced training, structured collaborative frameworks, and clear clinical pathways to ensure safe and effective practice. They also underscore the potential for pharmacist prescribers to reduce therapeutic inertia, improve access to timely medication adjustments, and alleviate physician workload benefits that align with international evidence from established prescribing models. Policymakers and healthcare leaders may use these results to guide the development of credentialing processes, interprofessional training programs, and supportive regulatory structures. Ultimately, empowering pharmacists and pharmacy students in training to build capacity in more efficient academic-healthcare delivery and improved cardiovascular outcomes.

The qualitative interviews conducted by a group of clinical pharmacists and a student carry inherent limitations related to subjectivity and positionality. Because the researchers are also practitioners, their interpretations are shaped by personal experiences, professional identity, and the specific clinical environment in which they work. This may limit the transferability of insights to other specialties, institutions, or healthcare systems with different prescribing cultures or scopes of practice. An additional limitation is that participants reflected on pharmacist prescribing not only from their own practice site but also from observations and experiences in external settings, conferences, or professional interactions. None of the participants held prescribing privileges at the time of the study, although institutional discussions were underway regarding the possibility of granting limited prescribing rights to selected clinical pharmacists. Because the study was autoethnography-informed, reflections were grounded in the lived experiences of the researchers rather than restricted to the study site alone. The dual role of researcher-participant introduces risks of selective recall, confirmation bias, and overemphasis on familiar or preferred practices. Furthermore, this collective autoethnography-informed approach relies heavily on introspection rather than external validation, meaning that the findings may not fully represent the perspectives of other stakeholders such as physicians, nurses, or patients. While this method provides depth, authenticity, and access to tacit dimensions of practice, these limitations should be acknowledged when considering the broader applicability of the results.

## 5 Conclusion

This study explored the perspectives of senior to junior clinical pharmacy professionals on the what, when, and why of pharmacist prescribing in cardiology as a continuing professional development. The study suggested a practice deeply rooted in clinical expertise, collaborative care, and a commitment to improving patient outcomes. Pharmacists described prescribing as a natural extension of their existing responsibilities, particularly in assessing patients, adjusting therapy, and initiating evidence-based medications for cardiovascular conditions. Their ability to prescribe was shaped by specialized training, interprofessional trust, and supportive practice environments. Motivations for prescribing centered on enhancing autonomy, reducing delays in care, and achieving meaningful improvements in patient outcomes. Together, these findings highlight the perceived role of pharmacist prescribing in optimizing cardiovascular therapy and strengthening multidisciplinary care models.

## Authors' contribution

SZ and DT conceptualized the study. DT designed the methodology. SZ, SN, AT, and SC contributed to data collection. SZ and DT analyzed the data, and SN, AT, SC, and SS validated the analysis. DT drafted the initial manuscript, and SZ and SS revised it. All authors read and approved the final manuscript.

## CRediT authorship contribution statement

**Seeba Zachariah:** Writing – review & editing, Visualization, Validation, Software, Methodology, Formal analysis, Data curation, Conceptualization. **Said Nabil:** Writing – review & editing, Validation, Data curation. **Anju Thomas:** Writing – review & editing, Validation, Data curation. **Selma Castell:** Writing – review & editing, Validation, Data curation. **Shatha Sayegh:** Writing – review & editing, Validation, Data curation. **Dixon Thomas:** Writing – review & editing, Writing – original draft, Supervision, Software, Project administration, Methodology, Formal analysis, Data curation, Conceptualization.

## Ethics approval and consent to participate

Research has been conducted in accordance with the Declaration of Helsinki. The Gulf Medical University Institutional Review Board approval was obtained for this study, IRB-COP-FAC-136-Mar-2025. Institutional permission was obtained from the site section head. Informed voluntary participation was given by the study participants. No patient or other health professional-level data or outcomes were included.

## Funding

No funding was received for conducting this research.

## Declaration of competing interest

None to disclose.

## Data Availability

Data are included in the article; additional data are available on request to the corresponding author.
